# Fibroblast growth factor 5 protects against spinal cord injury through activating AMPK pathway

**DOI:** 10.1111/jcmm.17934

**Published:** 2023-11-10

**Authors:** Feng Chen, Bing‐Rui Xiong, Shu‐Yue Xian, Jing Zhang, Rui‐Wen Ding, Ming Xu, Zong‐Ze Zhang

**Affiliations:** ^1^ Department of Anesthesiology Zhongnan Hospital of Wuhan University Wuhan China; ^2^ Department of Thoracic Surgery Zhongnan Hospital of Wuhan University Wuhan China

**Keywords:** AMP‐activated protein kinase, fibroblast growth factor 5, spinal cord injury

## Abstract

Excessive productions of inflammatory cytokines and free radicals are involved in spinal cord injury (SCI). Fibroblast growth factor 5 (FGF5) is associated with inflammatory response and oxidative damage, and we herein intend to determine its function in SCI. Lentivirus was instilled to overexpress or knockdown FGF5 expression in mice. Compound C or H89 2HCl were used to suppress AMP‐activated protein kinase (AMPK) or protein kinase A (PKA), respectively. FGF5 level was significantly decreased during SCI. FGF5 overexpression mitigated, while FGF5 silence further facilitated inflammatory response, oxidative damage and SCI. Mechanically, FGF5 activated AMPK to attenuate SCI in a cAMP/PKA‐dependent manner, while inhibiting AMPK or PKA with pharmacological methods significantly abolished the neuroprotective effects of FGF5 against SCI. More importantly, serum FGF5 level was decreased in SCI patients, and elevated serum FGF5 level often indicate better prognosis. Our study identifies FGF5 as an effective therapeutic and prognostic target for SCI.

## 1 INTRODUCTION

Spinal cord injury (SCI) is a severe traumatic event that eventually leads to motor, sensory and autonomic dysfunction.^1^ Inflammation and oxidative stress play critical roles in the pathogenesis of SCI.^2^, ^3^, ^4^ During SCI, resident inflammatory cells are activated, and subsequently generate various pro‐inflammatory cytokines, such as interleukin‐6 (IL‐6) and tumour necrosis factor‐α (TNF‐α). Meanwhile, the blood‐spinal cord barrier (BSCB) is ruptured, usually occurring within 5 min and lasting up to 28 days after SCI, which further facilitates leukocytes extravasation, inflammatory response and tissue damage.^5^ Moreover, these leukocytes also contribute to reactive oxygen species (ROS) overproduction, thereby provoking peroxidation of biomacromolecules (e.g. lipids, proteins and nucleic acids).^6^, ^7^ Thus, inhibiting inflammation and oxidative stress is vital for preventing SCI.

AMP‐activated protein kinase (AMPK), a multi‐functional serine/threonine protein kinase, is well‐known as a modulator of cellular energy metabolism; however, emerging studies have identify that it is also implicated in regulating inflammatory response and oxidative damage.^8^, ^9^, ^10^, ^11^ Findings from Hu et al.^12^ recently revealed that AMPK activation dramatically inhibited the phosphorylation and activation of nuclear factor‐κB (NF‐κB), thereby preventing inflammation in murine hearts. And Wang et al.^13^ found that AMPK activation facilitated the nuclear accumulation of nuclear factor E2‐related factor 2 (NRF2), and subsequently attenuated lipid peroxidation and diabetic cardiomyopathy. Consistently, various studies have shown that activating AMPK inhibits inflammation, oxidative stress and SCI in mice.^14^, ^15^ Moreover, our recent study also indicated that AMPK activation conferred a significant protection against SCI through controlling inflammatory response and ROS overproduction.^16^ Together, we conclude that targeting AMPK may provide novel approaches for the treatment of SCI.

Fibroblast growth factors (FGFs) are a family of heparin‐binding polypeptides and exhibit diverse biological functions.^7^, ^17^ Various FGFs have been shown to be associated with SCI. Vargas et al.^18^ found that FGF1 could activate haem oxygenase‐1 in a NRF2‐dependent manner, and subsequently prevent oxidative damage and neural apoptosis after SCI. And FGF2 treatment also significantly improved SCI‐induced locomotor dysfunction.^19^, ^20^ In addition, findings from Li et al.^21^ implied that FGF13 overexpression promoted neuronal polarization, axon formation and growth cone initiation, thereby facilitating functional recovery following SCI. FGF5 belongs to FGFs family, and is abundantly expression in nervous system, including the spinal cord.^22^, ^23^ Hanaka et al.^24^ demonstrated that FGF5 deficiency exacerbated inflammation and hepatic necrosis during non‐alcoholic steatohepatitis. Also, upregulating FGF5 dramatically decreased the expression of pro‐inflammatory cytokines and facilitated retinal ganglion cell survival.^25^ We herein intend to decipher the function of FGF5 in inflammatory response, oxidative damage and SCI in mice.

## 2 MATERIALS AND METHODS

### 2.1 Animals and treatments

Male C57BL/6 mice (10–12 week old) were housed in a specific pathogen free barrier system with free access to food and water for 1‐week adaptive feeding. To overexpress or knockdown FGF5 in the spinal cord, mice were intraspinally injected with 2 μL lentivirus (1 × 10^8^ TU/mL) carrying full‐length mouse FGF5 cDNA (NM_010203), short hairpin RNA against mouse FGF5 (shFGF5) or matched controls (Ctrl for FGF5, shCtrl for shFGF5) at rostral and caudal sites 3 mm from the lesion epicentre with approximately 0.5 mm in depth 2 weeks before SCI or sham surgery.^16^, ^26^ For AMPK or protein kinase A (PKA) inhibition, mice were intraperitoneally injected with 20 mg/kg compound C (CC, #S7840; Selleck Chemicals) or H89 2HCl (H89, #S1582; Selleck Chemicals) every 2 days 1 week pre‐SCI.^27^ Next, these mice were subjected to SCI surgery as we previously described.^16^ In brief, mice were anaesthetized and underwent a laminectomy at the T8 vertebral level. And then, the vertebral column was subjected to a 60 kdyn weight drop injury, and mice with the trembled body, stretched and turned legs and dropped tail were included as successful SCI models. Penicillin sodium solution and sterile saline were used for disinfection and fluid recovery. Meanwhile, sham‐operated mice received a same laminectomy with no injury to the vertebral column. According to the above information, all mice were assigned to five experiments, and the group information was provided as below. First experiment: Sham (*n* = 6), 1 days after SCI (*n* = 9), 3 days after SCI (*n* = 9), 7 days after SCI (*n* = 9), 14 days after SCI (*n* = 9), 28 days after SCI (*n* = 9). Second experiment: Sham + Ctrl (*n* = 8), Sham + FGF5 (*n* = 8), SCI + Ctrl (*n* = 10), SCI + FGF5 (*n* = 10). Third experiment: Sham + Ctrl (*n* = 8), Sham + FGF5 (*n* = 8), SCI + Ctrl (*n* = 10), SCI + FGF5 (*n* = 10). Fourth experiment: SCI + Ctrl + Vehicle (*n* = 10), SCI + FGF5 + Vehicle (*n* = 10), SCI + Ctrl + CC (*n* = 10), SCI + FGF5 + CC (*n* = 10). Fifth experiment: SCI + Ctrl + Vehicle (*n* = 10), SCI + FGF5 + Vehicle (*n* = 10), SCI + Ctrl + H89 (*n* = 10), SCI + FGF5 + H89 (*n* = 10). All protocols and animal care were approved and performed according to the guidelines of the Institutional Animal Care and Use Committee of Zhongnan Hospital of Wuhan University.

### 2.2 Clinical samples and ASIA scores

Serum samples were collected from SCI patients as previously described, and written informed consent was obtained from all participants.^28^ The study was approved by the Ethics Committee of Zhongnan Hospital of Wuhan University. Baseline information was collected and presented in Table 1. The American Spinal Injury Association (ASIA) scores were used to evaluate sensory and motor function after SCI by two independent physicians. In the ASIA sensory test, the sensory functions, including light touch and needle thorns feels, were evaluated by a clinical examination of 28 dermatomes each side of the limb, and both of the two sides were included. The sensory score was evaluated as follows: sensation is absent (score of 0), impaired (score of 1) or normal (score of 2), with a total sensory score of 112 = 28 (dermatomes) × 2 (score) × 2 (two sides) for light touch feel. Meanwhile, needle thorns feel also includes a score of 112. This means the total sensory score is 224. In addition, a total of 20 key muscles (10 key muscles in both upper limbs and 10 key muscles in both lower limbs) were used for the motor function analysis, and each muscle is scored from 0 to 5. This means the total score of the ASIA motor score is 100.^28^, ^29^

**TABLE 1:** Clinical characteristics of the study population.

	Ctrl (*n* = 56)	SCI (*n* = 92)
Age, years	53.7 ± 7.2	56.2 ± 5.4
Gender, male/female	34/22	55/37
Smoking, *n* (%)	25 (44.6)	34 (37.0)
Drinking, *n* (%)	31 (55.4)	46 (50.0)
Hypertension, *n* (%)	14 (25.0)	32 (34.8)
Diabetes, *n* (%)	10 (17.9)	17 (18.5)
Coronary heart disease, *n* (%)	8 (14.3)	15 (16.3)
Hyperlipidemia, *n* (%)	15 (26.8)	25 (27.2)

Abbreviations: Ctrl, control; SCI, spinal cord injury.

### 2.3 Sensory and motor assessments

Basso mouse scale (BMS) score, from 0 (no ankle movement) to 9 (normal gait), was calculated at indicating times to evaluate the hindlimb function, as we had previously described.^16^ Mechanical allodynia and thermal sensitivity were measured according to previous studies at 28 days after SCI, and experiments was performed without knowledge of the treatments.^16^, ^30^

### 2.4 BSCB permeability evaluation

To evaluate BSCB permeability, mice were intravenously injected with 2% Evans blue dye (#E2129; Sigma‐Aldrich) 28 days after SCI or sham surgery, maintained for an additional 3 h, and then were perfused to remove intravascular Evans Blue dye from the spinal cord. Subsequently, the spinal cord lesion was harvested, and tissue homogenate was mixed with 50% trichloroacetic acid solution. Following incubation at 60°C for 24 h and centrifugation at 10,000 *g* for 10 min, the fluorescence in supernatants was detected at 620 nm excitation and 680 nm emission using a spectrophotometer.^3^, ^16^

### 2.5 Quantitative real‐time PCR

Total RNA was extracted and reversely transcribed to cDNA following standard protocols as previously described by us and the others.^16^, ^31^ Next, quantitative real‐time PCR was performed on the LightCycler 480 system with SYBR Green Master Mix as the probe. Expression of mouse FGF5 mRNA was normalized to GAPDH mRNA using $2^{-∆∆C_{t}}$ formula, and primer sequences were as following: FGF5, forward, 5′‐AAGTAGCGCGACGTTTTCTTC‐3′ and reverse, 5′‐CTGGAAACTGCTATGTTCCGAG‐3′; GAPDH, forward, 5′‐AGGTCGGTGTGAACGGATTTG‐3′ and reverse, 5′‐TGTAGACCATGTAGTTGAGGTCA‐3′.

### 2.6 Western blot

Western blot was performed as previously described by us and the others.^16^, ^32^, ^33^ In brief, total proteins were extracted using RIPA lysis, quantified using Pierce™ BCA Protein Assay Kit (#23225; ThermoFisher Scientific) and separated using 10% SDS‐PAGE. Next, the proteins were transferred onto PVDF membranes, blocked in 5% skim milk and incubated with different primary antibodies overnight at 4°C. Primary antibodies included anti‐phospho‐NF‐κB p65 (p‐p65, #3033; Cell Signalling Technology), anti‐total p65 (t‐p65, #8242; Cell Signalling Technology), anti‐p‐AMPK (#2535; Cell Signalling Technology) and anti‐t‐AMPK (#5831; Cell Signalling Technology), anti‐glyceraldehyde‐3‐phosphate dehydrogenase (GAPDH, #ab8245; Abcam) and anti‐NRF2 (#ab62352; Abcam). After being washed for three times, the membranes were incubated with horseradish peroxidase‐conjugated secondary antibodies for 1.5 h at room temperature. Finally, the protein complexes were visualized by the electrochemiluminescence detection system using a ChemiDoc XRS+ Image System (Bio‐Rad) and analysed using the Image Lab software (Version 6.0).

### 2.7 Analysis of oxidative stress

ROS level in the spinal cord was analysed with 2′,7′‐dichlorofluorescin diacetate (DCFH‐DA, #D6883; Sigma‐Aldrich) as previously described by us and the others.^16^, ^34^, ^35^ In brief, the spinal cord homogenates were incubated with 50 μmol/L DCFH‐DA and for 30 min at 37°C with the fluorescent intensities detected at 504/524 nm. Meanwhile, levels of hydrogen peroxide and superoxide anion in the spinal cord were detected using Amplex™ Red Hydrogen Peroxide/Peroxidase Assay Kit (#A22188; ThermoFisher Scientific) and lucigenin (#M8010; Sigma‐Aldrich) according to the manufacturer's instructions. In addition, levels of malondialdehyde (MDA), 3‐nitrotyrosine (3‐NT) and 8‐hydroxy 2 deoxyguanosine (8‐OHdG) were detected using commercial kits (#ab118970 for MDA, #ab116691 for 3‐NT, #ab201734 for 8‐OHdG; Abcam) to evaluate oxidative damage of lipids, proteins and nucleic acids according to the manufacturer's instructions as we previously described.^16^

### 2.8 Biochemical analysis

FGF5 in mouse spinal cord and human serum samples were detected using Mouse FGF5 ELISA Kit (#CSB‐EL008632MO; CUSABIO) or Human FGF5 ELISA Kit (#CSB‐EL008632HU; CUSABIO), respectively according to the manufacturer's instructions. In brief, tissue homogenates or serum samples were incubated with 100 μL Biotin‐antibody for 1 h at 37°C and 100 μL HRP‐avidin for an additional 1 h at 37°C. Next, 90 μL TMB Substrate was added and allowed incubation for 30 min at 37°C in the dark, and then 50 μL Stop Solution was added. Finally, the optical density value was measured at 540 nm with a reference at 450 nm. To measure myeloperoxidase (MPO) activity in the spinal cord, mouse MPO ELISA Kit (#ab155458) was used as we previously described.^16^ In brief, tissue homogenates were prepared and added into appropriate wells to incubate overnight at 4°C. Next, the samples were incubated with 1 × biotinylated antibody for 1 h and streptavidin solution for 45 min at room temperature, followed by an incubation with TMB One‐Step Substrate Reagent for 30 min in the dark. Finally, Stop Solution was added, and the optical density value was measured at 450 nm. To measure NF‐κB or NRF2 transcription activity, nuclear extracts were prepared and incubated with the TransAM® NF‐κB p65 Kit (#40096; Active Motif) or TransAM® NRF2 Kit (#50296; Active Motif). Levels of IL‐6 and TNF‐α in the spinal cord were detected using mouse IL‐6 ELISA Kit (#M6000B; R&D Systems) and TNF‐α ELISA Kit according to the manufacturer's instructions. Levels of cyclic AMP (cAMP) and PKA in the spinal cord were detected using a competitive cAMP Assay Kit (#ab65355; Abcam) and PKA Kinase Activity Assay Kit (#ab139435; Abcam) as previously described.^36^

### 2.9 Statistical analysis

Data are analysed by SPSS software (version 23.0) and presented as the mean ± standard deviation. Unpaired Student's *t*‐test was applied to compare differences between two groups, and one‐way anova with the Tukey post‐hoc test was used to compare differences among three or more groups. *p* < 0.05 was established statistical significance.

## 3 RESULTS

### 3.1 FGF5 overexpression alleviates SCI in mice

**FIGURE 1:**
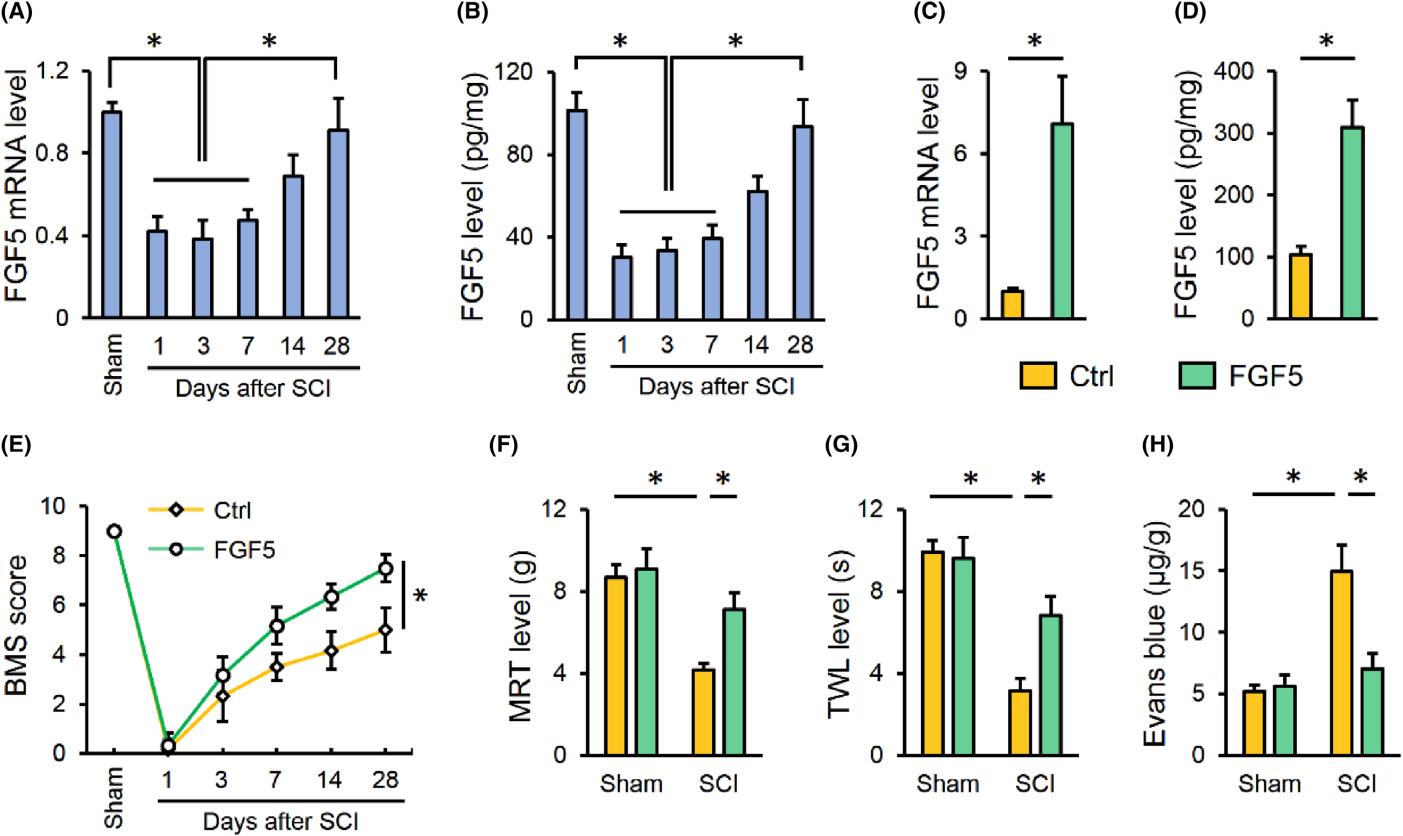
FGF5 overexpression prevents SCI in mice. (A) Mice were exposed to Ctrl or SCI surgery, and FGF5 mRNA level in the spinal cord was detected at indicating times. (B) FGF5 level was detected using an ELISA kit. (C, D) Mice were intraspinally injected with 2 μL lentivirus carrying FGF5 to overexpress FGF5 in the spinal cord, and FGF5 mRNA and protein levels were detected 2 weeks post‐injection. (E) Mice were overexpressed with FGF5 using lentiviral vectors and then were exposed to SCI or sham surgery 2 weeks post‐injection. BMS score, from 0 (no ankle movement) to 9 (normal gait), was determined at indicating times. (F, G) Sensitivities to mechanical and thermal stimulation were determined 28 days after SCI. (H) Extravasation of Evans blue dye was determined 28 days after SCI. *n* = 6 for each groups. Data are expressed as the mean ± standard deviation and *p* < 0.05 was considered statistically significant. *p < 0.05.

To detect the role of FGF5, FGF5 level post‐SCI was measured. As shown in Figure 1A,B, FGF5 expression in the spinal cords was significantly reduced after SCI, with a peak between Day 1 to Day 7. Subsequently, the expression of FGF5 was gradually restored, even to the basal levels at Day 28. Next, mice were overexpressed with FGF5 using a lentiviral system (Figure 1C,D). FGF5 overexpression prevented SCI‐induced motor dysfunction in mice, as evidenced by an increased BMS score (Figure 1E). Meanwhile, FGF5 overexpression restored mechanical response threshold (MRT) and thermal withdrawal latency (TWL) in SCI mice, confirming that FGF5 attenuated SCI‐induced mechanical and thermal hypersensitivity (Figure 1F,G). In addition, SCI‐induced disruption of BSCB integrity was significantly improved by FGF5 overexpression (Figure 1H). Collectively, we demonstrate that FGF5 overexpression alleviates SCI in mice.

### 3.2 FGF5 silence exacerbates SCI in mice

**FIGURE 2:**
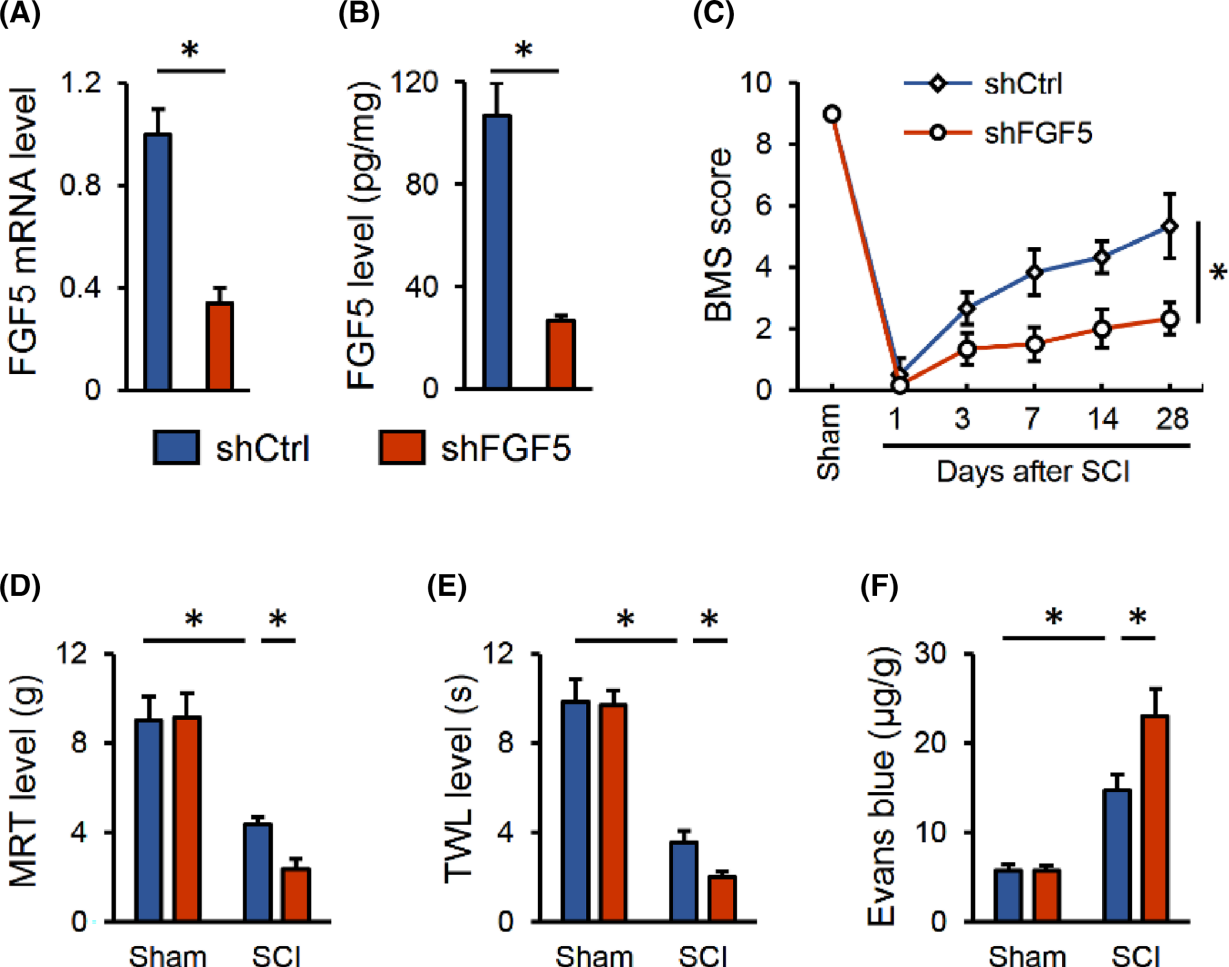
FGF5 knockdown exacerbates SCI in mice. (A, B) Mice were intraspinally injected with 2 μL lentivirus carrying shFGF5 to knockdown FGF5 in the spinal cord, and FGF5 mRNA and protein levels were detected 2 weeks post‐injection. (C) Mice were injected with shFGF5, and then were exposed to SCI or sham surgery 2 weeks post‐injection. BMS score, from 0 (no ankle movement) to 9 (normal gait), was determined at indicating times. (D, E) Sensitivities to mechanical and thermal stimulation were determined 28 days after SCI. (F) Extravasation of Evans blue dye was determined 28 days after SCI. *n* = 6 for each groups. Data are expressed as the mean ± standard deviation and *p* < 0.05 was considered statistically significant. *p < 0.05.

Next, lentiviral vectors carrying shFGF5 were used to silence FGF5 in mice (Figure 2A,B). As shown in Figure 2C, BMS score was further decreased in SCI mice with FGF5 silence, indicating a worse motor function. And SCI‐induced hypersensitivity to mechanical and thermal stimulations were also aggravated by FGF5 silence (Figure 2D,E). Moreover, Evans blue dye leakage to the tissue was more severe in FGF5‐silenced mice after SCI (Figure 2F). Collectively, we determine that FGF5 silence exacerbates SCI in mice.

### 3.3 FGF5 overexpression reduces inflammatory response and oxidative stress in SCI mice

**FIGURE 3:**
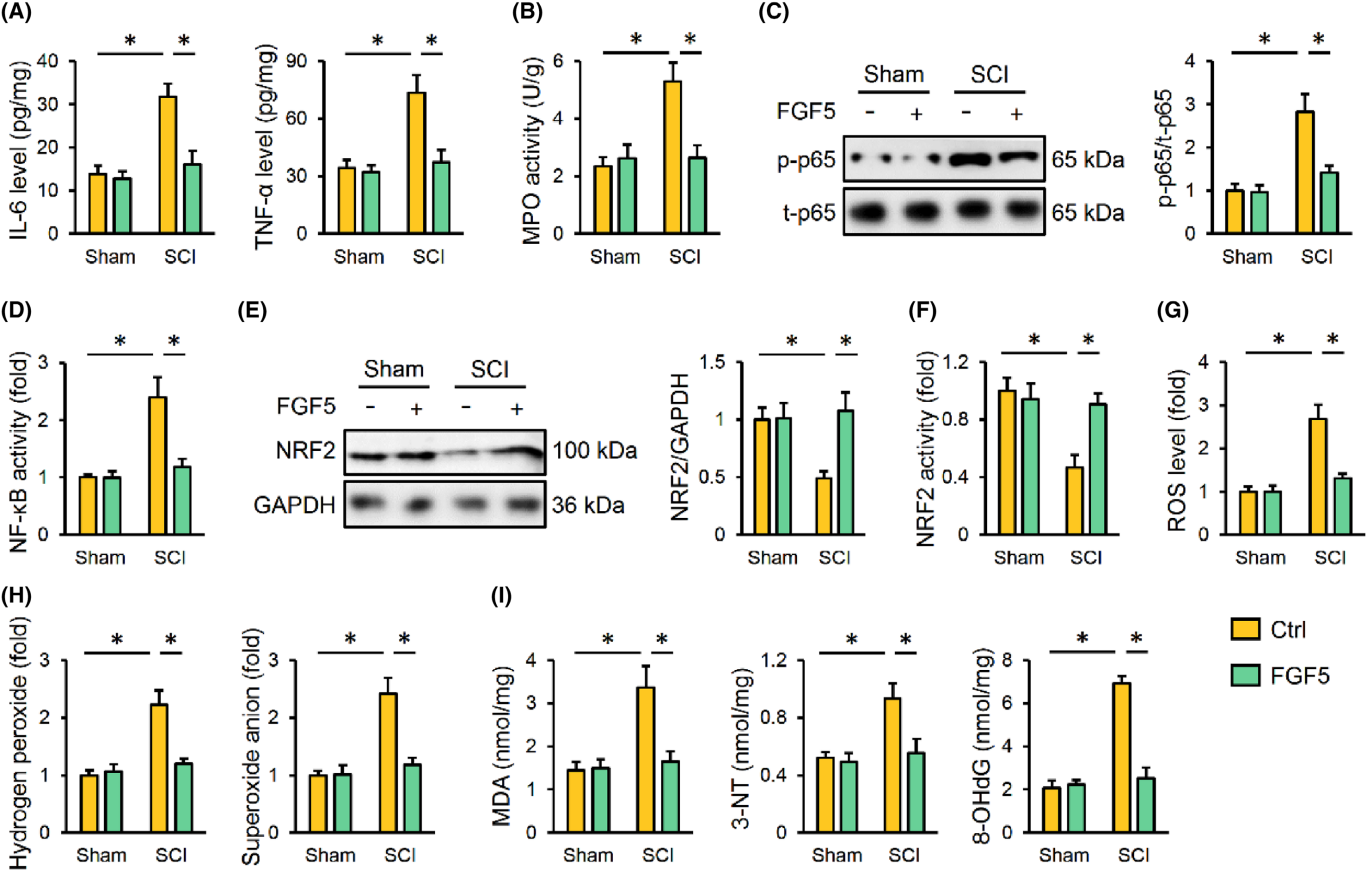
FGF5 overexpression reduces inflammation and oxidative stress in SCI mice. (A) Mice were overexpressed with FGF5 using lentiviral vectors, and then were exposed to SCI or sham surgery 2 weeks post‐injection. IL‐6 and TNF‐α levels in the spinal cord were detected. (B) MPO activity in the spinal cord. (C) Western blot images and quantification of p65 phosphorylation. (D) Relative NF‐κB transcription activity. (E, F) Relative levels of NRF2 protein and transcription activity. (G) ROS level detected by DCFH‐DA probe. (H) Quantification of hydrogen peroxide and superoxide anion in spinal cord. (I) The levels of MDA, 3‐NT and 8‐OHdG. *n* = 6 for each groups. Data are expressed as the mean ± standard deviation and *p* < 0.05 was considered statistically significant. *p < 0.05.

Excessive productions of inflammatory cytokines and free radicals are involved in SCI; therefore, we evaluated the effects of FGF5 in SCI‐induced inflammatory response and ROS generation. Interestingly, SCI‐induced elevations of IL‐6 and TNF‐α were reduced by FGF5 overexpression (Figure 3A). Meanwhile, FGF5 overexpression also suppressed MPO activity in SCI mice, indicating that FGF5 overexpression decreased neutrophils accumulation (Figure 3B). NF‐κB, a nodal transcription factor in triggering inflammation, was significantly activated by SCI, which was then inhibited in those with FGF5 overexpression (Figure 3C,D). NRF2 is essential for the transcription of various anti‐oxidant genes, and activating NRF2 helps to scavenging excessive free radicals in SCI mice. As shown in Figure 3E,F, the expression and activity of NRF2 were decreased upon SCI stimulation, which were significantly restored by FGF5 overexpression. Accordingly, SCI‐induced overproductions of free radicals were effectively decreased in FGF5‐overexpressed mice (Figure 3G,H). Meanwhile, oxidative damage to biomacromolecules in SCI mice was prevented by FGF5 overexpression, as evidenced by decreased MDA, 3‐NT and 8‐OHdG levels (Figure 3I). Collectively, we demonstrate that FGF5 overexpression reduces inflammatory response and oxidative stress in SCI mice.

### 3.4 FGF5 knockdown elevates inflammatory response and oxidative stress in SCI mice

**FIGURE 4:**
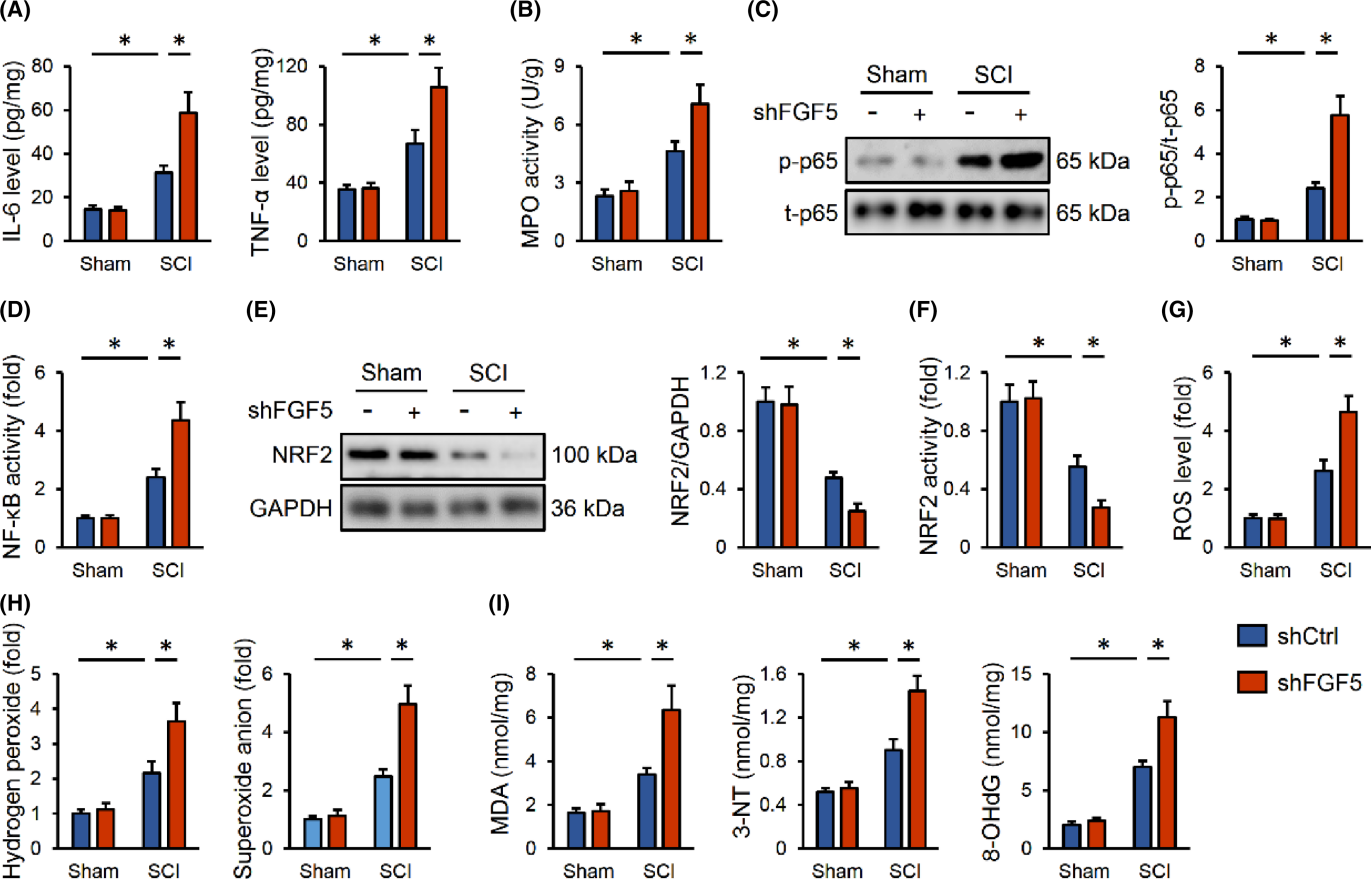
FGF5 knockdown elevates inflammation and oxidative stress in SCI mice. (A) Mice were injected with shFGF5, and then were exposed to SCI or sham surgery 2 weeks post‐injection. IL‐6 and TNF‐α levels in the spinal cord were detected. (B) MPO activity in the spinal cord. (C) Western blot images and quantification of p65 phosphorylation. (D) Relative NF‐κB transcription activity. (E, F) Relative levels of NRF2 protein and transcription activity. (G) ROS level detected by DCFH‐DA probe. (H) Quantification of hydrogen peroxide and superoxide anion in spinal cord. (I) The levels of MDA, 3‐NT and 8‐OHdG. *n* = 6 for each groups. Data are expressed as the mean ± standard deviation and *p* < 0.05 was considered statistically significant. *p < 0.05.

As expected, the elevations of IL‐6 and TNF‐α in SCI mice were further increased by FGF5 silence (Figure 4A). And FGF5 silence also augmented MPO activity in SCI mice (Figure 4B). Consistently, the phosphorylation and activity of NF‐κB were further enhanced in FGF5‐silenced SCI mice (Figure 4C,D). Meanwhile, SCI‐induced inhibitions on NRF2 expression and transcription activity were also enhanced by FGF5 silence (Figure 4E,F). Consistent with the impairment of NRF2 activity, FGF5 silence further facilitated the accumulations of free radicals in the spinal cord post‐SCI (Figure 4G–I). Together, we determine that FGF5 silence elevates inflammatory response and oxidative stress in SCI mice.

### 3.5 FGF5 overexpression reduces inflammation, oxidative stress and SCI through activating AMPK

**FIGURE 5:**
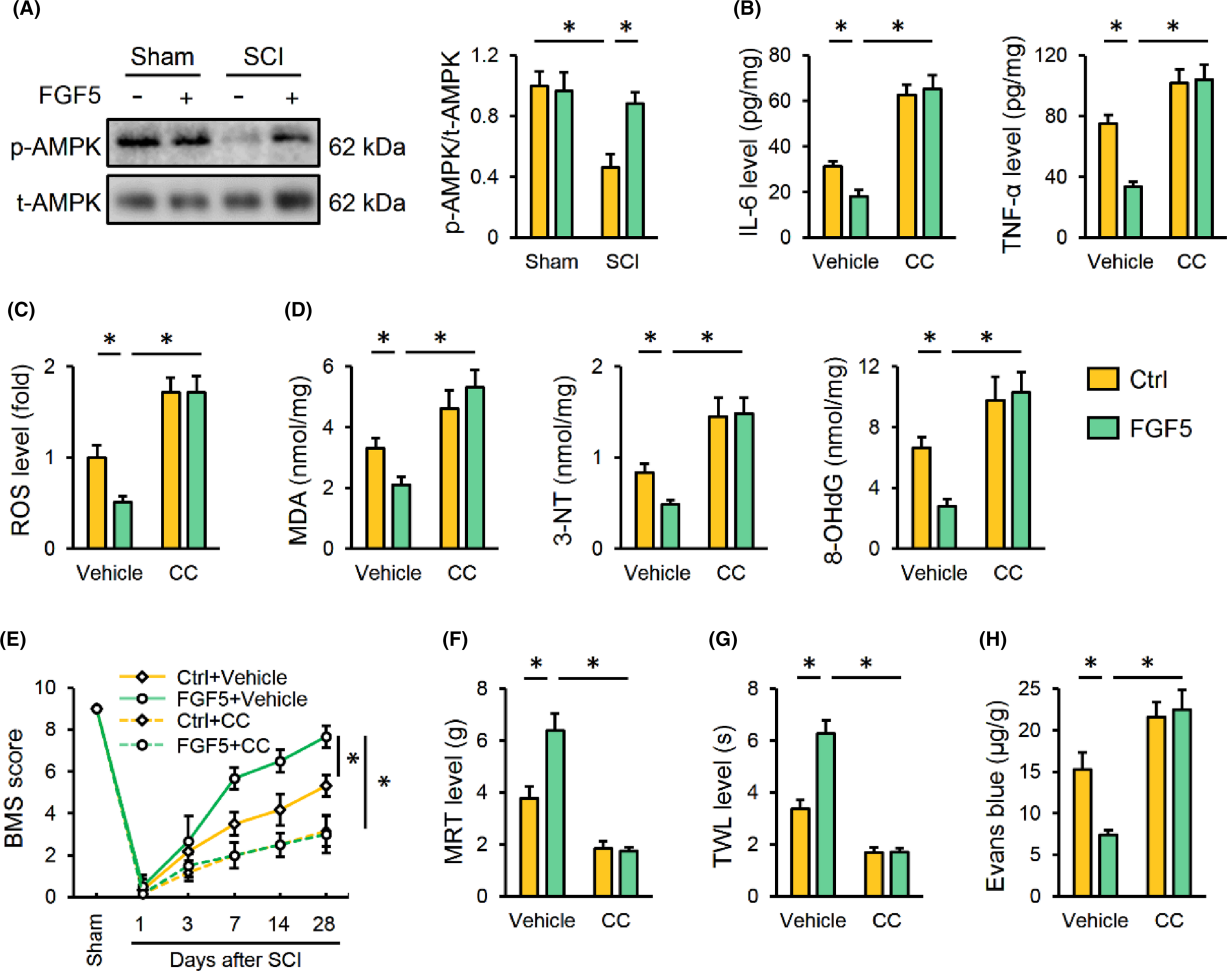
FGF5 overexpression reduces inflammation, oxidative stress and SCI through activating AMPK. (A) Mice were overexpressed with FGF5 using lentiviral vectors, and then were exposed to SCI or sham surgery 2 weeks post‐injection. Western blot images and quantification of AMPK phosphorylation in the spinal cord were detected. (B) To inhibit AMPK, mice with or without FGF5 overexpression were intraperitoneally injected with CC every 2 days 1 week pre‐SCI. IL‐6 and TNF‐α levels in the spinal cord were detected. (C) ROS level detected by DCFH‐DA probe. (D) The levels of MDA, 3‐NT and 8‐OHdG. (E) BMS score, from 0 (no ankle movement) to 9 (normal gait), was determined at indicating times. (F, G) Sensitivities to mechanical and thermal stimulation were determined 28 days after SCI. (H) Extravasation of Evans blue dye was determined 28 days after SCI. *n* = 6 for each groups. Data are expressed as the mean ± standard deviation and *p* < 0.05 was considered statistically significant. *p < 0.05.

AMPK is essential for regulating inflammation and oxidative damage, and we recently also proved that activating AMPK significantly attenuated SCI in mice.^16^, ^37^, ^38^ Therefore, we investigated whether FGF5 overexpression reduced inflammation, oxidative stress and SCI through AMPK. Interestingly, SCI‐induced suppression on AMPK was significantly restored by FGF5 overexpression (Figure 5A). Next, CC was used to inhibit AMPK in SCI mice to clarify its necessity. As expected, CC injection significantly abolished the prohibitive effects of FGF5 on SCI‐induced inflammatory response and oxidative damage, as indicated by increased IL‐6, TNF‐α, ROS, MDA, 3‐NT and 8‐OHdG levels (Figure 5B–D). Consistent with the molecular alterations, FGF5 overexpression‐mediated protection against SCI‐induced motor dysfunction was remarkably blocked by AMPK inhibition, as evidenced by a decreased BMS score (Figure 5E). Meanwhile, the beneficial effects of FGF5 on mechanical and thermal hypersensitivity in SCI mice were diminished in those with CC injection (Figure 5F,G). CC treatment also abolished the prohibitory function of FGF5 on BSCB disruption in SCI mice (Figure 5H). Collectively, we demonstrate that FGF5 overexpression reduces inflammation, oxidative stress and SCI through activating AMPK.

### 3.6 FGF5 overexpression activates AMPK through cAMP/PKA pathway

**FIGURE 6:**
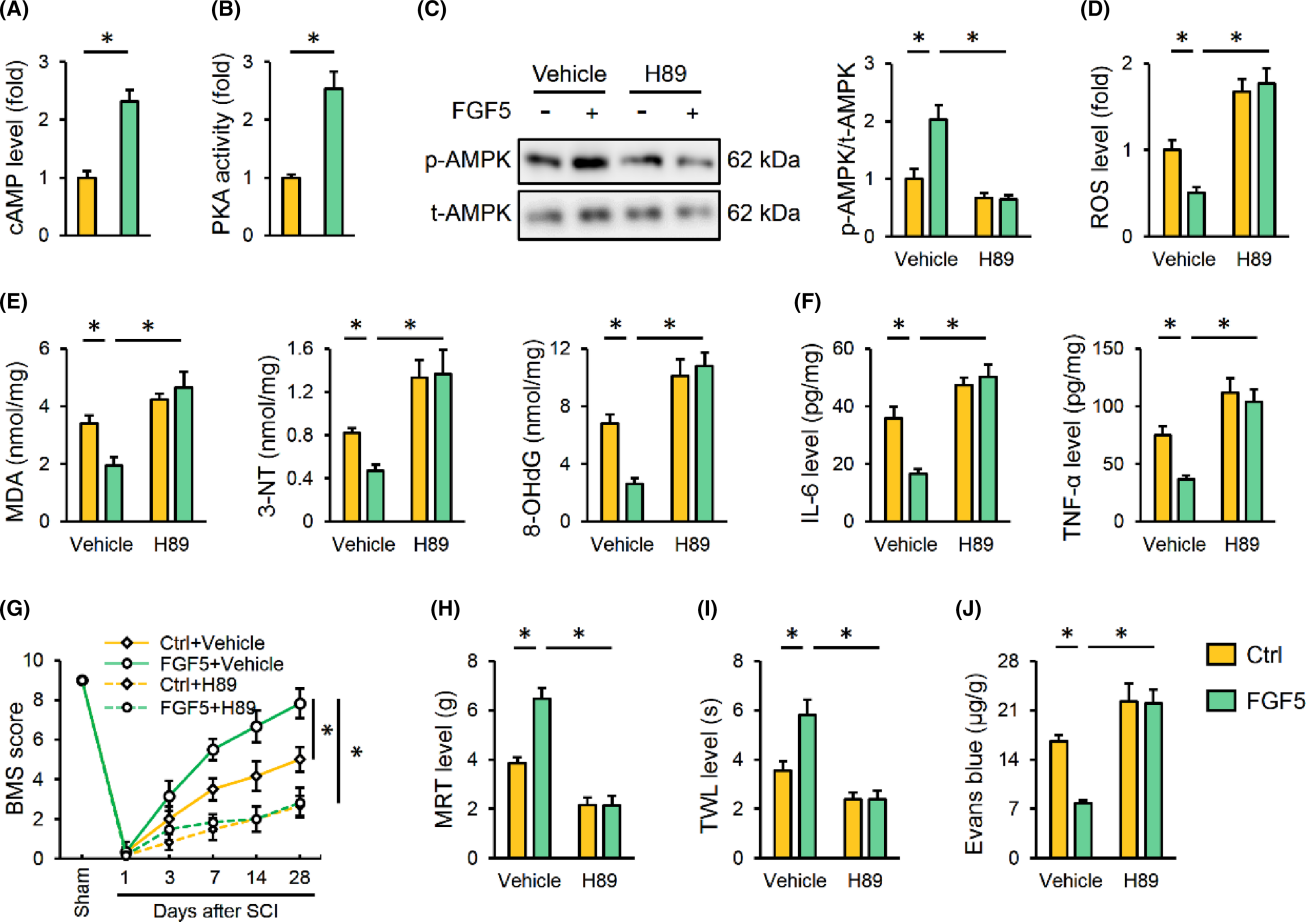
FGF5 overexpression activates AMPK through cAMP/PKA pathway. (A, B) Mice were intraspinally injected with 2 μL lentivirus carrying FGF5 to overexpress FGF5 in the spinal cord, and cAMP level and PKA activity were detected 2 weeks post‐injection. (C) To inhibit PKA, mice with or without FGF5 overexpression were intraperitoneally injected with H89 every 2 days 1 week pre‐SCI. Western blot images and quantification of AMPK phosphorylation in the spinal cord were detected. (D) ROS level detected by DCFH‐DA probe. (E) The levels of MDA, 3‐NT and 8‐OHdG. (F) IL‐6 and TNF‐α levels in the spinal cord were detected. (G) BMS score, from 0 (no ankle movement) to 9 (normal gait), was determined at indicating times. (H, I) Sensitivities to mechanical and thermal stimulation were determined 28 days after SCI. (J) Extravasation of Evans blue dye was determined 28 days after SCI. *n* = 6 for each groups. Data are expressed as the mean ± standard deviation and *p* < 0.05 was considered statistically significant. *p < 0.05.

Subsequently, we explored the underlying mechanism through which FGF5 activates AMPK. Second messengers are essential for transducing extracellular stimuli into intracellular pathways, among which, cAMP acts upstream of the AMPK pathway.^39^ As shown in Figure 6A, FGF5 overexpression significantly elevated cAMP levels in the spinal cord. PKA is a classic downstream mediator to induce AMPK activation by cAMP.^40^ Interestingly, we found that FGF5 overexpression also activated PKA in the spinal cord (Figure 6B). To investigate whether FGF5 activates AMPK through PKA, H89 was used to suppress PKA. Undoubtedly, FGF5 failed to activate AMPK in the presence of H89 after SCI (Figure 6C). H89 treatment also significantly blocked FGF5‐mediated suppressions on SCI‐induced oxidative stress and inflammation (Figure 6D–F). Consistently, CC treatment also abrogated FGF5 overexpression‐associated protections against SCI‐induced motor dysfunction, mechanical and thermal hypersensitivity (Figure 6G–I). BSCB damage was also aggravated in FGF5‐overexpressed SCI mice by PKA inhibition (Figure 6J). Collectively, we demonstrate that FGF5 overexpression activates AMPK through cAMP/PKA pathway.

### 3.7 Serum FGF5 level positively correlates with the sensory and motor function in SCI patients

**FIGURE 7:**
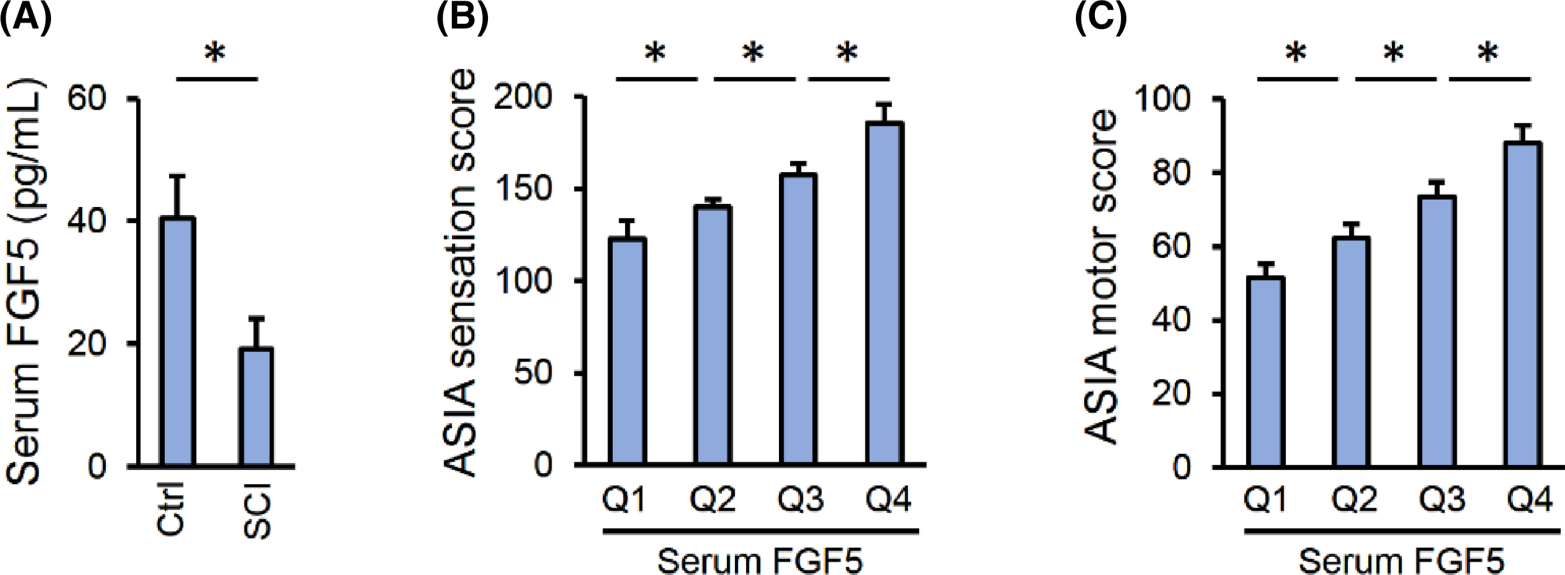
Serum FGF5 level positively correlates with the sensory and motor function in SCI patients. (A) Serum levels of FGF5 in Ctrl and SCI patients. (B, C) SCI patients were divided into four groups according to serum FGF5 levels, and ASIA sensation and motor scores were measured at indicating groups. *n* = 56 for Ctrl group and *n* = 92 for SCI group. Data are expressed as the mean ± standard deviation and *p* < 0.05 was considered statistically significant. *p < 0.05.

Considering the neuroprotective effects of FGF5 against SCI in mice, we finally investigated whether serum FGF5 could be a prognostic marker for SCI patients. The baseline information was provided in Table 1, and no difference in baseline parameters between the groups. Yet, serum FGF5 level was significantly reduced in SCI patients (Figure 7A). We next divided patients into 4 groups according to the quartile range of serum FGF5 levels, and analysed their ASIA scores. As shown in Figure 7B,C, the two ASIA scores were increased with the elevation of serum FGF5 levels. Collectively, we demonstrate that serum FGF5 level positively correlates with the sensory and motor function in SCI patients.

## 4 DISCUSSION

SCI is an irreversible neurotrauma, and no effective methods are available to treat SCI. We herein revealed that FGF5 level was significantly decreased during SCI. FGF5 overexpression mitigated, while FGF5 silence further facilitated inflammatory response, oxidative damage and SCI in mice. Mechanically, FGF5 activated AMPK to attenuate SCI in a cAMP/PKA‐dependent manner, while inhibiting AMPK or PKA with pharmacological methods significantly abolished the neuroprotective effects of FGF5 against SCI. More importantly, serum FGF5 level was decreased in SCI patients, and elevated serum FGF5 level often indicate better prognosis. Our study identifies FGF5 as an effective therapeutic and prognostic target for SCI.

BSCB disruption is an early response after SCI, and predisposes the infiltration of peripheral immune cells to the lesions. Maikos and Shreiber^5^ previously demonstrated that BSCB was disrupted immediately as early as 5 min after SCI. Then, circulating leukocytes are recruited to the lesions to amplify the inflammatory response and oxidative damage in the spinal cord. Neutrophils are the primary inflammatory cells to be assembled during acute phase of SCI.^41^ Subsequently, they produce massive inflammatory cytokines that elicit a chronic inflammatory response and neurological dysfunction. In addition, recruited neutrophils also enhance the generations of proteases and free radicals, thereby facilitating lipid peroxidation, DNA fragmentation, membrane damage and cellular injury.^42^ In our study, we determined that BSCB disruption and neutrophils accumulation in SCI mice was attenuated by FGF5 overexpression, but further exacerbated by FGF5 knockdown. Accordingly, FGF5 overexpression significantly reduced inflammation and oxidative damage in SCI mice. NRF2 functions as a central transcription factor of redox homeostasis, and its activation is essential for the expression of many anti‐oxidant enzymes, such as superoxide dismutase (SOD), catalase (CAT), and so forth.^43^ NRF2 exists in the cytoplasm by interacting with Kelch‐1ike ECH‐associated protein 1 (KEAP1), and then exposed to ubiquitination‐mediated degradation under physiological conditions. Upon ROS stimulation, NRF2 detaches from KEAP1 and enters to the nucleus to increase the expression of SOD and CAT.^44^ SOD catalases superoxide anion to generate hydrogen peroxide, which is then catabolized by CAT to form water and oxygen. Accordingly, pioneering experiments from us and the others have determined that inhibiting inflammation and oxidative damage provide significant neuroprotection against SCI.^16^, ^41^

Most studies about FGF5 are mainly focused on embryonic development and tumorigenesis. Haub and Goldfarb^45^ previously demonstrated that FGF5 was expressed in post‐implantation epiblast, lateral splanchnic mesoderm, lateral somatic mesoderm, myotomes, mastication muscle, limb mesenchyme and acoustic ganglion in the developing mouse embryo, and played critical roles in murine development. Subsequent studies found that FGF5 was abundantly expressed in neural tissues, including the spinal cord.^22^, ^23^ Yet, no experiment has been performed to investigate its role in SCI. Herein, we revealed that FGF5 level was decreased in the spinal cord of SCI mice and serum samples in SCI patients. FGF5 overexpression attenuated, while FGF5 silence further facilitated inflammatory response, oxidative damage and SCI. In addition, we determined that FGF5 overexpression elevated cAMP level in the spinal cord and then activated the PKA/AMPK pathway. Moreover, serum FGF5 levels positively correlates with the sensory and motor function in SCI patients.

## 5 CONCLUSION

In summary, we identify FGF5 as an effective therapeutic and prognostic target for SCI.

## AUTHOR CONTRIBUTIONS

**Feng Chen:** Data curation (equal); methodology (equal); software (equal); validation (equal). **Bing‐Rui Xiong:** Conceptualization (equal); data curation (equal); methodology (equal); software (equal); validation (equal); writing – original draft (equal); writing – review and editing (equal). **Shu‐Yue Xian:** Data curation (equal); formal analysis (equal); methodology (equal); resources (equal). **Jing Zhang:** Methodology (equal); software (equal); validation (equal); visualization (equal). **Rui‐Wen Ding:** Methodology (equal); validation (equal); visualization (equal). **Ming Xu:** Conceptualization (equal); investigation (equal); project administration (equal); writing – original draft (equal); writing – review and editing (equal). **Zong‐Ze Zhang:** Conceptualization (equal); investigation (lead); writing – original draft (equal); writing – review and editing (equal).

## CONFLICT OF INTEREST STATEMENT

The authors declare that there are no conflicts of interests.

## Data Availability

The data that support the findings of this study are available from the corresponding author upon reasonable request.
